# Reduction of pathological retinal neovascularization, vessel obliteration, and artery tortuosity by PEDF protein in an oxygen‐induced ischemic retinopathy rat model

**DOI:** 10.1096/fba.2024-00059

**Published:** 2024-07-19

**Authors:** Shiying Zhao, Alexander V. Tschulakow, Subha S. Karthikeyan, Kun Wang, Stefan Kochanek, Ulrich Schraermeyer, Sylvie Julien‐Schraermeyer

**Affiliations:** ^1^ Division of Experimental Vitreoretinal Surgery, Centre for Ophthalmology, Institute for Ophthalmic Research University Medical Center, Eberhard Karls University of Tuebingen Tuebingen Germany; ^2^ OcuTox GmbH Preclinical Drug Assessment Hechingen Germany; ^3^ Department of Gene Therapy University Clinic Ulm Germany; ^4^ Present address: Molecular mechanisms driving age‐related macular degeneration, Experimental Vitreoretinal Surgery Group Centre for Ophthalmology, Institute for Ophthalmic Research, University Medical Center, Eberhard Karls University of Tuebingen Tuebingen Germany

**Keywords:** angiogenesis, artery tortuosity, OIR rat model, PEDF protein therapy, ROP, vessel obliteration

## Abstract

Retinopathy of prematurity (ROP) is a severe retinal disease in premature infants characterized by pathological neovascularization, obliteration of retinal vessels and increased vessel tortuosity. Currently, there are no completely satisfactory treatments for ROP. Pigment epithelium‐derived factor (PEDF), a potent inhibitor of angiogenesis, appears late in gestation and its deficiency may be linked to development of ROP. This study investigates the preclinical efficacy of PEDF protein alone or in combination with VEGF antagonists for treating ROP. The safety of PEDF protein in the rat eye was assessed using functional in vivo measurements and histology. The efficacy of intravitreal injections (IVI) of various treatments was evaluated in a rat oxygen‐induced retinopathy (OIR) model using in vivo imaging and flatmount analyses. No functional or histological side‐effects were found in rat eyes after intravitreal PEDF protein injection. PEDF protein alone or combined with anti‐VEGF drugs significantly reduced pathological neovascularization and vessel obliteration, comparable to the effects of anti‐VEGF drugs alone. Regarding arterial tortuosity, treatment with a combination of PEDF, and VEGF antagonist was more effective than treatment with anti‐VEGF alone. IVI of PEDF protein is safe. PEDF protein alone or combined with VEGF antagonists shows similar efficacy in reducing pathological neovascularization and vessel obliteration as anti‐VEGF agents. Furthermore, only treatments involving PEDF protein, alone or with VEGF antagonists, significantly improved the quality of retinal vasculature. Thus, PEDF protein alone or combined with anti‐VEGF agents presents a promising alternative to current anti‐VEGF treatments for ROP.

## Graphical abstract

PEDF protein alone or combined with VEGF antagonists shows similar efficacy in reducing pathological neovascularization and vessel obliteration as anti‐VEGF agents. But, only treatments involving PEDF protein, alone or with VEGF antagonists, significantly improved the quality of retinal vasculature by reducing artery tortuosity, and were therefore more effective than treatment with anti‐VEGF alone. Thus, PEDF protein alone or combined with anti‐VEGF agents presents a promising alternative to current anti‐VEGF treatments for ROP.

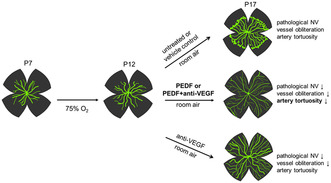

## 1 INTRODUCTION

Premature babies are often placed in incubators with high oxygen saturation,^1^ a necessary measure for survival but one that presents a dilemma between resuscitation and the physical development of preterm infants. According to the BOOST II clinical trial, increased oxygen saturation in incubators can lead to reduced severe cerebral functions, retinopathy of prematurity (ROP), and even death.^1^, ^2^, ^3^

ROP is a serious disease that can cause severe visual impairment, blindness, and consequently a decline in the quality of life in infants.^2^, ^4^, ^5^, ^6^ It is characterized by abnormal retinal vasculature including changes in tortuosity, avascular areas (AV), and areas with pathological neovascularization which can eventually lead to retinal detachment.^7^ The precise pathophysiology of ROP is not completely elucidated; however the widely accepted theory is the two‐phase theory^7^, ^8^: Phase I begins with the initiation of infant breathing, where the oxygen saturation of the developing retina in the premature baby is relatively higher than in the intrauterine state, leading to attenuated production of vascular endothelial growth factor (VEGF) in the retina, suspending vascularization. High oxygen incubation exacerbates VEGF suppression. During phase I, the retina endures relative hyperoxia and incomplete vascularization. In phase II, the avascular area of the retina suffers from ischemia and secretes an abundant amount of VEGF, resulting in disorganized retinal vascular growth.

Treatments for ROP have improved over the past four decades. In the 1980s, cryotherapy was the standard treatment for ROP.^9^ It has been proved to reduce the severity of the ROP according to the CRYO ROPCG trial. In the 2000s, clinical trials demonstrated that laser ablation therapy for ROP surpassed cryotherapy in effectiveness.^10^, ^11^, ^12^ Both cryotherapy and laser ablation aim to sacrifice the avascular peripheral retina to reduce complications that may compromise the central retina.^13^ However, these treatments do not preserve the function of the avascular retina, potentially leading to long‐term visual field loss during infant development. Additionally, cryotherapy and laser ablation can cause severe and irreversible complications such as intraocular hemorrhage and cataracts.^14^ Since 2007, anti‐VEGF therapy has been used to treat ROP. Both the RAINBOW and BEAT‐ROP clinical trials showed that anti‐VEGF therapy is more effective than laser ablation.^15^, ^16^ The intravitreal injection (IVI) of anti‐VEGF therapeutics is less traumatic to the eye and easier to perform. However, the disadvantages of anti‐VEGF therapy, such as delayed vascularization, recurrence, challenging follow‐up and particularly unknown long‐term effects, make its use controversial.^15^, ^17^, ^18^, ^19^, ^20^ Therefore, there is still a high medical need for efficient and safe treatments for ROP patients.

Pigment epithelium‐derived factor (PEDF), naturally expressed in eye tissues,^21^, ^22^ appears late in gestation and its absence may contribute to ROP development upon transition from high‐to‐ambient oxygen environments or with intermittent hypoxia.^23^ PEDF is a promising candidate for ROP treatment as it is a natural antagonist of VEGF^24^ and a potent inhibitor of angiogenesis, as demonstrated in several animal models of ocular angiogenesis.^23^, ^25^, ^26^, ^27^, ^28^, ^29^ Recently, our group discovered that PEDF protein can suppress pathological changes in vessels by keeping the capillary lumen open, leading to enhanced survival of neural retinal cells in an ex vivo ischemia model^30^ and of photoreceptors in a choroidal neovascularization (CNV) rat model (Tschulakow et al., 2023).^31^ This study investigates the effect of PEDF protein therapy alone or in combination with two anti‐VEGF drugs (bevacizumab, an anti‐human VEGF antibody, and an anti‐rat VEGF^164^ antibody). We compared the efficacy of these treatments to that of an untreated OIR control, a PBS control, and VEGF antagonists alone in our established oxygen‐induced ischemic retinopathy (OIR) rat model, simulating ROP.^28^ In this model, rat pups were first exposed to high oxygen conditions causing the retinas to adapt. Returning the pups to room air led to hypoxia in the developing retinal tissues resulting in a disturbed VEGF/PEDF balance and typical pathological retinal vascular changes. This study examines whether the disturbed VEGF/PEDF ratio can be balanced by a single IVI of PEDF protein alone or in combination with VEGF antagonists. The study focuses on angiogenesis parameters and the quality of retinal vasculature combining in vivo imaging (scanning laser ophthalmoscopy/fluorescein angiography/optical coherence tomography [SLO/FA/OCT]) with flatmount analysis. Additionally, the in vivo retinal safety of PEDF protein in the rat eye was evaluated using functional in vivo measurements and histology.

## 2 MATERIALS AND METHODS

### 2.1 Ethics statement

This study utilized experiments conducted on rats. All experiments were performed with the approval of the Animal Experimentation Committee of the University of Tuebingen “Regierungspraesidium Tuebingen” (AK 01/21G). All animals were handled in accordance with the German Animal Welfare Act and were under the control of the animal protection agency and supervision of veterinarians at the Eberhard Karls University of Tuebingen. The study did not involve any human experiments.

### 2.2 Animals

For the ocular safety study, 52 adult Long Evans rats from Janvier Labs (Le Genest‐Saint‐Isle, France) were used.

For the OIR study, four pregnant Long Evans rats (Janvier Labs) were used, resulting in a total of 43 pups. These were allocated to the dose range study (12 pups) and the subsequent study (31 pups). Each mother rat and her pups were kept in a separate cage.

### 2.3 Safety study in rat eyes

Both eyes of adult Long Evans rats were injected intravitreally with 5 μL of PEDF protein solutions. The maximum possible dose of PEDF protein (100 μg/eye) and two additional doses, each reduced by a factor of 5 (i.e., 20 and 4 μg/eye) were used and investigated at four different time points (day 1, day 3, day 7, and day 14) after IVI using histology and functional assessment through electroretinography (ERG). Four rats (eight eyes) served as untreated controls (no PEDF, day 0) and four rats (eight eyes) were used per PEDF dose (4 μg, 20 μg, and 100 μg per eye) and per time point (day 1, day 3, day 7, and day 14) (Table 1).

**TABLE 1:** Summary of the rat and eye number per PEDF dose and time point used for the safety study.

PEDF dose and time point	Number of rats (eyes)
Untreated control: no PEDF—day 0	4 (8)
PEDF 4 μg—day 1	4 (8)
PEDF 4 μg—day 3	4 (8)
PEDF 4 μg—day 7	4 (8)
PEDF 4 μg—day 14	4 (8)
PEDF 20 μg—day 1	4 (8)
PEDF 20 μg—day 3	4 (8)
PEDF 20 μg—day 7	4 (8)
PEDF 20 μg—day 14	4 (8)
PEDF 100 μg—day 1	4 (8)
PEDF 100 μg—day 3	4 (8)
PEDF 100 μg—day 7	4 (8)
PEDF 100 μg—day 14	4 (8)

#### 2.3.1 IVI

A small incision was made into the conjunctiva at the outer corner of the eyes. The eyeball was rotated by grasping the conjunctiva with a pair of fine tweezers and gently pulling. A volume of 5 μL was injected intravitreally through the hole using a 10 μL NanoFil syringe with a NanoFil 34‐gauge beveled needle (World Precision Instruments). After the injection, the needle remained in the eye for an additional 3–4 s to reduce reflux and was then drawn back. The eyeball was brought back into its normal position, and antibiotic ointment was applied to the eye. The whole procedure was performed using a surgical microscope equipped with illumination.

#### 2.3.2 Processing of the eyes

On days 0, 1, 3, 7, and 14, the animals were sacrificed. The eyes were then fixed in 4.5% formalin (Roti Histofix, Carl Roth, Karlsruhe, Germany) and embedded in paraffin according to standard procedures.

#### 2.3.3 Histopathological and inflammation assessments

Hematoxylin eosin (HE) stained sections were investigated for changes in the morphology of the retina and vitreous. The number of infiltrating cells within the vitreous was counted in paraffin sections under a light microscope at a magnification of 200×, corresponding to an area of 0.89 mm^2^. If there were more than 100 infiltrating cells in this area, the number was set at 100.

Mueller cell activation was estimated using an anti‐glial fibrillary acidic protein (GFAP) mouse monoclonal antibody (Cell Signaling Technology, Inc. Los Angeles, USA) on sagittal retinal sections. Three neighboring sections were stained from the left eyes of all rats. The activation of Mueller cells was estimated using the following scoring system:

**FIGURE 1:**
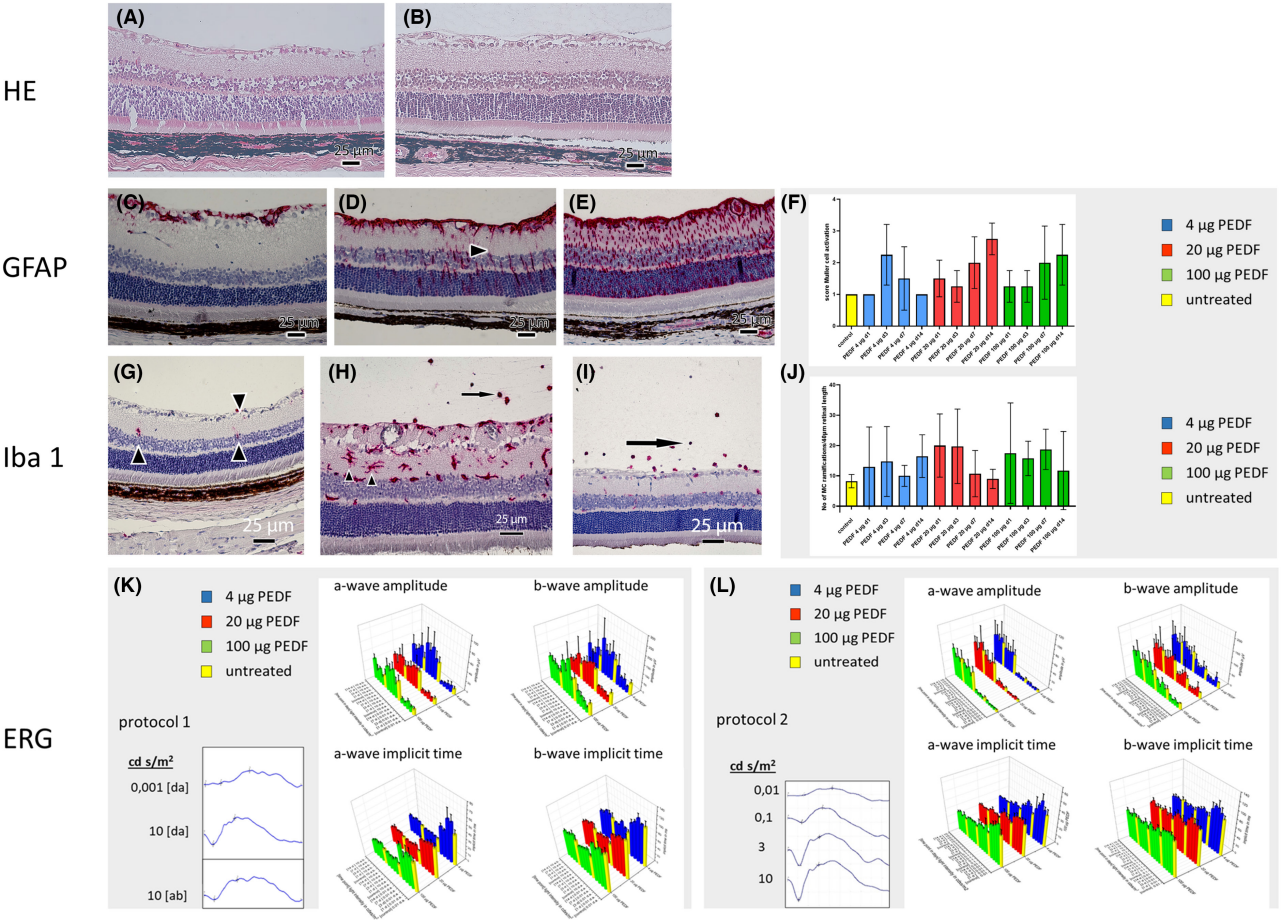
Safety/toxicity study in rat eyes. (A, B) HE staining 1 day (A) and 14 days (B) after intravitreal injection of 100 μg PEDF protein. No alterations were observed. (C–F) GFAP staining (red), (C) A representative image of Mueller cell activation's score 1 from an untreated control eye: GFAP staining is present only in the Mueller cell end feet. (D) A representative image of Mueller cell activation's score 2 from a retina 7 days after injection of 100 μg PEDF: GFAP is expressed locally by Mueller cells from the inner limiting membrane to the outer limiting membrane (arrowhead). (E) A representative image of Mueller cell activation's score 3 14 days after injection of 20 μg PEDF: Increased expression of GFAP by Mueller cells throughout the section (F) Quantification of Mueller cell activation by scoring of GFAP expression. (G–J) Iba‐1 staining (red), (G) Untreated control eye with resident microglia cells (MC) located within the inner plexiform layer and the ganglion cell layer (arrowheads). (H) Retina of a rat 3 days after injection of 20 μg PEDF with numerous activated microglia cells indicated by the development of dendrites (arrowheads) and infiltrating Iba‐1 positive cells in the vitreous (arrow). (I) Retina of a rat 3 days after injection of 100 μg PEDF showing fewer activated Iba‐1 positive cells within the retina compared to (H) but numerous infiltrating Iba1 cells in the vitreous (arrow). (J) Quantification of the number of Iba‐1 positive cells with elongated ramified processes per 40 μm retinal length. (K, L) ERG results, (K) Lower left panel: Representative electroretinogram for protocol 1 after dark adaptation [ad]: With stimuli of 0.001 cd. s/m^2^ and 10 cd. s/m^2^ and after bleaching (20 s, 10 cd. s/m^2^) [ab] with a 10 cd. s/m^2^ stimulus. On the right panel the results for all a‐wave and b‐wave amplitude and implicit time measurements for the doses 4, 20, and 100 μg PEDF/eye at the time points 1, 3, 7, and 14 days after injection and their corresponding controls are shown as 3D mean and SD plots. No significant changes after any of the treatments at any of the analyzed time points compared to the corresponding controls were found. (L) Lower left panel: Representative electroretinogram for protocol 2, ERGs for increasing stimulus intensities of 0.01, 0.1, 3, and 10 cd. s/m^2^. On the right panel the results for all a‐wave and b‐wave amplitude and implicit time measurements for the doses 4, 20, and 100 μg PEDF/eye at the time points 1, 3, 7, and 14 days after injection and their corresponding controls are shown as 3D mean ± SD plots. No significant changes after any of the treatments at any of the analyzed time points compared to the corresponding controls were found.

1 = GFAP staining only around retinal ganglion cells (as shown in Figure 1C).

2 = Mueller cells containing GFAP from the inner limiting membrane (ILM) to the outer limiting membrane (OLM) locally (as shown in Figure 1D).

3 = Mueller cells containing GFAP from ILM to OLM throughout the section (as shown in Figure 1E).

Microglia cells/macrophages (MC) were stained using an anti‐Ionized calcium binding adaptor molecule (Iba‐1) rabbit antibody (Wako Chemicals USA).

Glial cells within the retina were counted under a light microscope in sections from the left eyes of each rat at a magnification of 400× over the retinal length of 40 μm/field. If several dendrites were cut in an area about 50 μm in diameter, they were regarded as originating from only one MC.

#### 2.3.4 ERG

The animals were dark‐adapted overnight before ERG measurements. The ERGs were measured in 27 animals. The rats were anesthetized by an intraperitoneal injection of three‐component narcosis (0.005 mg fentanyl, 2 mg midazolam, and 0.15 mg of medetomidine/kg body weight). The pupils were dilated with 1–2 drops of a mydriatic agent (Mydriatikum, Pharmacy of the University of Tuebingen, Germany). Gold ring electrodes (1.5 mm diameter), (Roland Consult, Stasche & Finger GmbH, Brandenburg, Germany) were placed on the cornea of both eyes. Methocel (OmniVision, Puchheim, Germany) eye drops were used for coupling and to prevent drying of the eyes. Subdermal platinum (27 gauge) needles (Technomed Europe, Maastricht, The Netherlands) in the forehead between the eyes and at the base of the tail served as reference and ground electrodes, respectively. The light stimuli were delivered in a Ganzfeld dome (Roland Consult, Brandenburg, Germany). The ERG‐response amplitudes were measured using two automated standard protocols.

For the first protocol, dark‐adapted responses were elicited by brief flashes of white light on a dark background. Two stimulus intensities were used, a low one (0.001 cd. s/m^2^) to analyze the rod function and a high one (10 cd. s/m^2^) to analyze the mixed rod‐cone function. After that, the eyes were bleached (20 s, 10 cd. s/m^2^) and reanalyzed (10 cd. s/m^2^). For the second protocol, measurements for increasing stimulus intensities (0.01, 0.1, 3, 10 cd. s/m^2^) were performed. The RETI system software (Roland Consult, Brandenburg, Germany) was used for recording and analysis of the ERG data. The electroretinograms were corrected by a system‐internal 50 Hz filter for background suppression. After that, the a‐wave and b‐wave amplitudes and implicit times were measured on the electroretinograms.

### 2.4 OIR rat model

#### 2.4.1 Study design

**FIGURE 2:**
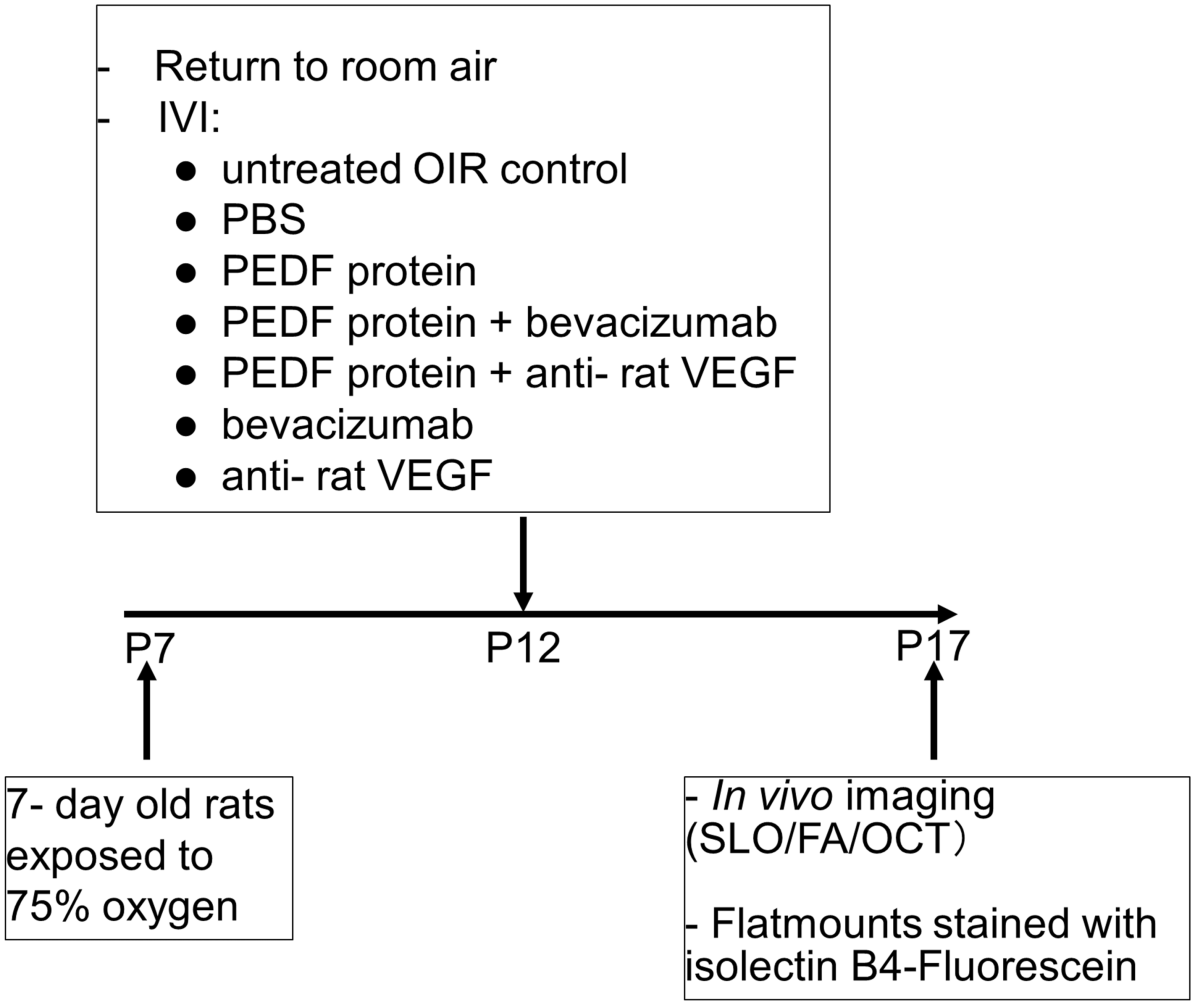
OIR study design. The rat pups were exposed to 75% oxygen from P7 to P12, then returned to room air. Immediately thereafter, they received an intravitreal injection of the different treatments in both eyes under isoflurane anesthesia. On postnatal day 17 (P17), the rats were investigated in vivo using SLO OCT. Then the rats were euthanized and enucleated. Flatmounts were prepared and stained with Griffonia Simplicifolia Lectin I (GSLI) isolectin B4‐Fluorescein. IVI, Intravitreal injection.

From postnatal day 7 (P7) to P12, the mother rats and their pups were exposed to 75% oxygen in an incubator (Biospherix, Ltd., Parish, NY, USA), then returned to room air. After a short adaptation time of 2 , the pups received IVI of the different treatments in both eyes (Figure 2, Table 2) under isoflurane anesthesia.^28^ After that, the animals were kept in normal air conditions for another 5 days until the time point of the in vivo and final analyses.

**TABLE 2:** Summary of the rat and eye numbers and the different treatments used for the OIR studies.

	Untreated OIR control	PBS	PEDF	PEDF	PEDF	PEDF + bevacizumab	PEDF + anti‐rat VEGF	Bevacizumab	Anti‐rat VEGF
Number of rats	7	6	6	2	2	5	3	6	6
Number of eyes	14	12	12	4	4	10	6	12	12
Treatment	–	2 μL	6.125 μg in 2 μL	12.25 μg in 2 μL	24.5 μg in 2 μL	(PEDF 6.125 μg + bevacizumab 25 μg) in 2 μL	(PEDF 6.125 μg + anti‐rat VEGF 25 ng) in 2 μL	25 μg in 2 μL	25 ng in 2 μL

Abbreviation: PBS, Phosphate buffered saline.

#### 2.4.2 IVI

The injections were performed under 3% isoflurane narcosis. Additionally, a local anesthetic, Novesine (OmniVision, Puchheim, Germany) was applied to the eyes. For the IVI, NanoFil Syringes (WPI, Sarasota, USA) with NanoFil 34‐gauge beveled needles were used. 2 μL of the corresponding substance solution were delivered through the *pars plana* into the vitreous cavity in each eye.^32^ After the injections, the eyes were treated with an antibiotic ointment, Bepanthen (Bayer Leverkusen, Germany) to prevent ocular infection.

#### 2.4.3 Substances

PBS (Gibco, Life Technologies limited, UK); bevacizumab, an anti‐human VEGF monoclonal antibody (25 mg/mL, Roche, Basel, Switzerland) and an anti‐rat VEGF^164^ (0.2 mg/mL, AF564, Bio‐Techne®R&D Systems, MN, USA) were used.

Recombinant human PEDF protein was produced in CHO cells using an expression vector encoding a full length human PEDF protein. The expressed protein was secreted into the culture supernatant and purified using chromatographic techniques. The purified human PEDF protein was used alone or in combination with anti‐rat or anti‐human VEGF drugs.

#### 2.4.4 PEDF protein dose range finding study

To determine the optimal dose of human PEDF protein that significantly inhibited retinal pathological neovascularization and vessel obliteration, four eyes per dose of PEDF protein were intravitreally injected on P12 the day of transition from high‐to‐normal oxygen with doses of 24.5 μg, 12.25 μg, and 6.125 μg PEDF in 2 μL PBS. As we already demonstrated the efficacy of 10 μg of PEDF protein in an ex vivo ischemia model^30^ and in a CNV rat model (Tschulakow et al., 2023),^31^ we decided to test also the dose of about 10 μg/eye (12.25 μg were used due to the batch's concentration) and two additional doses corresponding to the double and the half. The effects of the different doses on neovascularization, vessel obliteration and hyaloid vessels were assessed at P17.

#### 2.4.5 In vivo SLO/OCT imaging and measurement of the vessel diameters on OCT images

SLO, FA, and OCT were conducted using a Spectralis™ HRA + OCT device (Heidelberg Engineering, Heidelberg, Germany). To adapt the device for use in rats, a + 78D double aspheric lens (Volk Optical, Inc., Mentor, OH 44060, U.S.A.) was placed directly on the device's outlet. Additionally, a custom‐made contact lens with + 7 diopters (dpt) was placed directly onto the cornea of the animals immediately before analysis. On P17, the rat pups were intraperitoneally injected with a three‐component narcosis (fentanyl 0.005 mg/kg, midazolam 2 mg/kg and medetomidine 0.15 mg/kg) and their pupils were fully dilated using 1–2 drops of mydriatikum (from the Pharmacy of the University of Tuebingen, Germany). Subsequently, methocel (Omni Vision GmbH, Germany) was applied to prevent corneal drying and ensure better adherence of the + 7 dpt lens on the eye. To ensure proper positioning during the in vivo examination, the rats were placed on an adjustable platform directly in front of the device. Then 0.15 mL of 10% Fluorescein (Fluorescein Alcon; 1/10 dilution in isotonic 0.9% NaCl solution [Fresenius, Germany]) was subcutaneously injected. Immediately afterward, the FA of each eye was performed. The device was centered on the optic nerve head (ONH) and images were acquired. Subsequently, OCT analysis of each eye was conducted. OCT‐scans were manually taken in the area of the first bifurcations of the vessels. The diameters of the veins and arteries were measured on these images using the “Heidelberg Eye Explorer” software provided by the manufacturer (Heidelberg Engineering, Heidelberg, Germany).

#### 2.4.6 Flatmount preparation and staining of the vessels

After the in vivo examinations, the pups were euthanized, and their eyes were enucleated. Flatmounts were prepared following the protocol described by Tual‐Chalot et al.,^33^ with some slight adjustment, that is, the retina and the choroid were not separated to reduce the risk of tearing the retina.

The vessels were stained with Griffonia Simplicifolia Lectin I (GSLI) isolectin B4‐Fluorescein (Vector Laboratories, CA, USA) following standard protocols provided by the manufacturer. Subsequently, the flatmounts were mounted using Dako Fluorescence Mounting Medium (Agilent Technologies, Inc.CA, USA). Scan images covering the entire retina were generated from each flatmount using an Axio Imager Z1 microscope (ZEISS, Germany).

#### 2.4.7 Quantification of avascular area and neovascular area on flatmounts

The protocol published by Vähätupa and colleagues^34^ was followed. In brief, using the Image J software (Java 13.0.6, http://imagej.nih.gov/ij), the whole area of each flatmount (WA) as well as the AV and the neovascular areas (NV) were selected manually using the freehand drawing tool. Areas on flatmounts or whole flatmounts that were not analyzable due to preparation artifacts were excluded from the analysis. The neovascularization area (% of the whole area) and avascular area (% of the whole area) were calculated accordingly. For statistical analyses, the groups were compared to each other using ANOVA.

#### 2.4.8 Quantification of the vessel tortuosity on flatmounts

The veins were distinguished from the arteries as described in Pannarale et al.^35^ The arteries near the ONH are positioned over the central veins. Using Image J, the length of all veins and arteries from the ONH to their first bifurcation point as well as the distance between these two points were measured (Figure 4A,B). Tortuosity was defined as the ratio of the vessel length from the ONH to their first bifurcation point to the distance between the ONH and the first bifurcating point.

$$\text{Tortuosity}=\frac{\text{length of the vessel from the}\;\mathrm{ONH}\;\text{to their first bifurcation point}}{\text{distance between}\;\mathrm{ONH}\;\text{and the first bifurcation point}.}$$

#### 2.4.9 Quantification of the vessel diameter on flatmounts

The diameters of the veins and arteries were measured at the point of the first bifurcation. Measurements were taken perpendicular to the vessel walls.

### 2.5 Statistics and graphing

Prism 8.0 (GraphPad Software, San Diego, California USA, www.graphpad.com) was employed for statistical analyses. The results are presented as means ± standard deviation (SD). For statistical analyses involving multiple groups, ANOVA for multiple comparisons was utilized. Student's *t*–test was employed for the analysis of two groups. Statistical significance was considered at a *p* value <0.05. The results of the ERG analyses were graphed as 3D plots using ORIGIN PRO software (Version 2024 10.1).

## 3 RESULTS

### 3.1 Safety study in rat eyes

#### 3.1.1 Light microscopical findings

The HE stained images of the retina did not show any alterations at the different time points, even at the highest PEDF dose used (100 μg/eye) (Figure 1A,B). However, regardless of the PEDF dose used, infiltrating cells were detected within the vitreous 3 days after PEDF injection. Seven and 14 days after PEDF injection, infiltrating cells were only rarely observed, and there was no dose–response relationship in the number of infiltrating cells at the different PEDF concentrations used (data not shown).

#### 3.1.2 Mueller and microglia cell/macrophage activation

GFAP, a marker for Mueller cells, is up regulated in aging and under conditions of retinal damage or stress. In untreated control eyes, GFAP expression was limited to the end feet of the Müller cells near the vitreo‐retinal interface (Figure 1C–E). Examples of different activation scores are depicted in Figure 1C–E. Mild GFAP activation was observed 1–3 days after PEDF injection with more prominent activation after 3 days at the lowest concentration (4 μg PEDF). Treatment with 20 and 100 μg PEDF, led to increased GFAP expression throughout the observation period peaking at day 14. At a dose of 4 μg PEDF, there was a sustained decrease in GFAP expression from day three to day 14, and the Mueller cells returned to their non‐activated state by day 14 (Figure 1F). No changes in the shape of the Mueller cells or activation of astrocytes were observed.

In untreated control eyes, resident MC were situated within the inner plexiform layer and ganglion cell layer (Figure 1G). Following PEDF injection, MC became activated, evident from the enhanced development of dendrites (Figure 1H), termed hyper‐ramification. There was high intra‐individual variation in the number of activated cells across all treatment groups, with no significant differences observed. The number of cells with elongated ramified processes in the sections was slightly increased after PEDF treatment. Immunocytochemical staining revealed that nearly all infiltrating cells within the vitreous were positive for Iba‐1, indicating they were MC or macrophages (Figure 1I). 1–3 days after PEDF injection, there was no difference in the number of retinal MC among the different concentrations (Figure 1J). By day 14 after treatment with 20 and 100 μg PEDF, the number of elongated ramified processes had already decreased and was similar to that observed in untreated retinae.

#### 3.1.3 ERG measurements

No functional changes were observed following the injection of 4, 20, or 100 μg PEDF at days 1, 3, 7, and 14 after injection compared to the corresponding controls (Figure 1K,L). This finding corroborates the absence of retinal alterations observed in the HE stained images (Figure 1A,B).

### 3.2 PEDF protein dose range finding study in the rat OIR model

**FIGURE 3:**
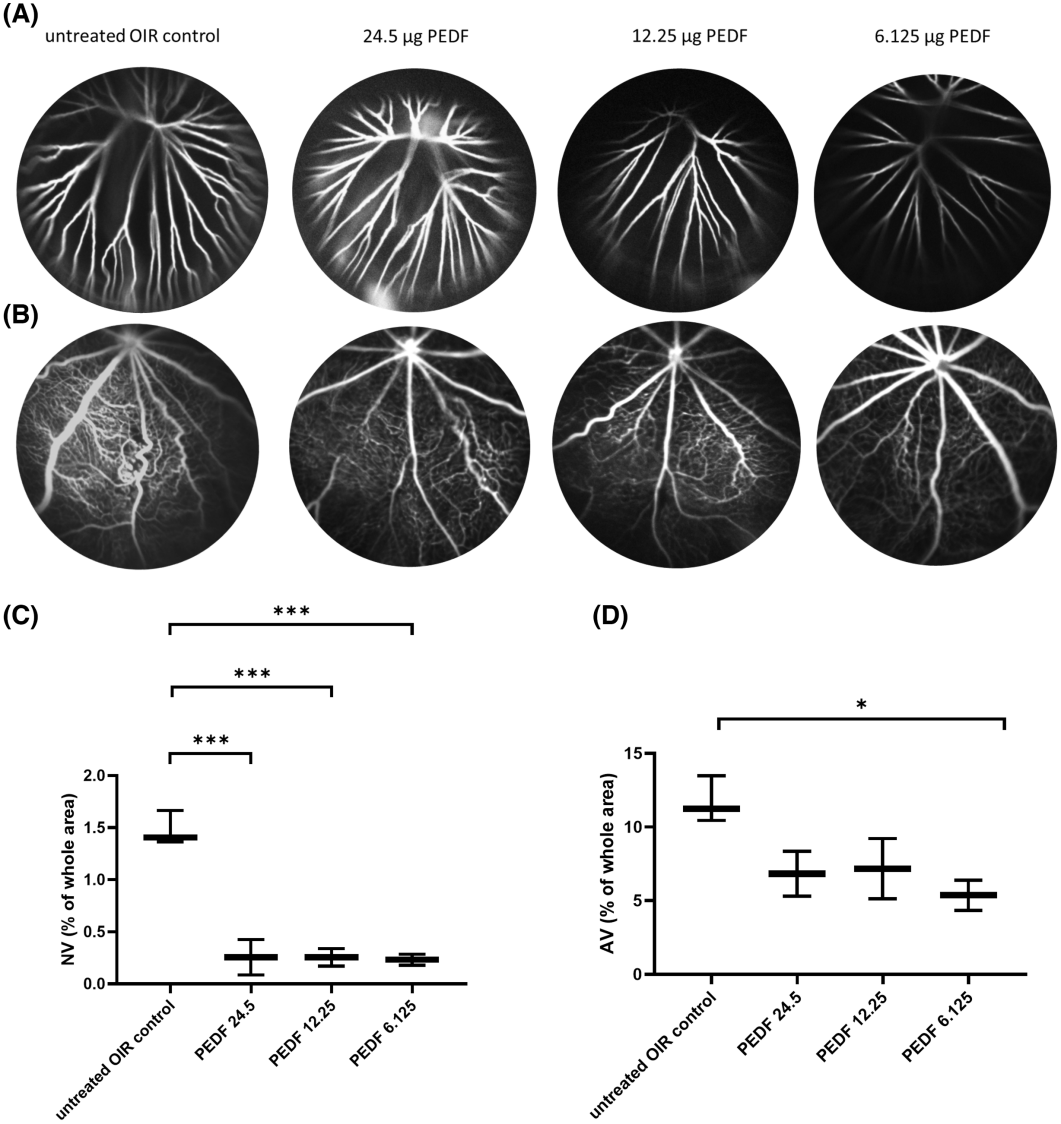
PEDF protein dose range finding study. (A) FA/SLO images of hyaloid vessels and (B) of the retinal vessels, of P17 rat eyes with OIR. Images of an untreated eye and eyes treated with 24.5 μg, 12.25 μg, and 6.125 μg PEDF respectively are shown. (C, D) ANOVA analysis of the areas with neovascularization (C) and avascular areas (AV) (D) on flatmounts as a percentage of the whole area are presented. The principle of the analysis is described under material and methods and indicated in Figure 4C. Mean and standard deviation are shown, *p < 0.05, ***p < 0.0001.

Representative in vivo results of the PEDF protein dose range finding study are depicted in Figure 3. The analysis revealed that treatment with 6.125 μg PEDF exhibited less pronounced hyaloid vessels (Figure 3A) and vessel density and tortuosity (Figure 3B) compared to the other groups, closely resembling animals growing under normoxic conditions, as shown in.^34^ Further analysis of the flatmounts demonstrated that all three PEDF doses significantly suppressed neovascularization (Figure 3C, ANOVA, ***p < 0.0001), whereas only PEDF at a dose of 6.125 μg/eye significantly reduced the avascular area compared to the untreated OIR control (Figure 3D, ANOVA, *p < 0.05). Overall, the dose of 6.125 μg PEDF protein/eye exhibited the most beneficial effects on the eyes following OIR and was consequently utilized in the subsequent experiments.

### 3.3 Investigation of the treatments with PEDF protein alone or in combination with anti‐VEGF agents in the rat OIR model

#### 3.3.1 Analysis of the pathological retinal neovascularization and vessel obliteration

**FIGURE 4:**
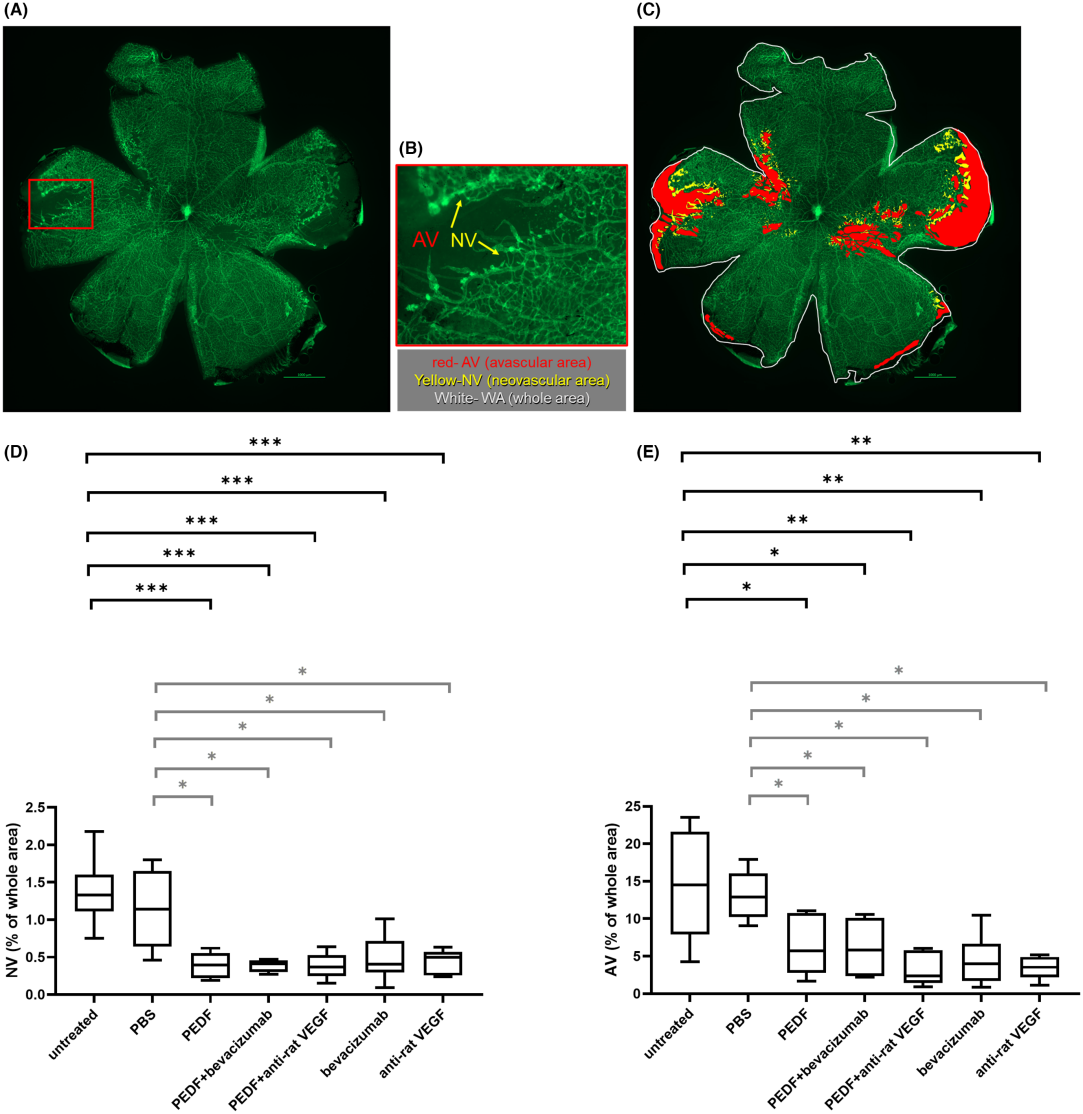
Analysis of the neovascularization and vessel obliteration on flatmount images. (A) Representative image of a flatmount of a P17 rat eye with OIR. An area with pronounced vessel obliteration and neovascularization is outlined in red. (B) A magnified image of the area framed in red in (A). (C) Principle of the analysis: The avascular (red) and neovascularization (yellow) areas were manually selected on the flatmount images. (D, E) ANOVA analysis of the areas with neovascularization (D) and avascular areas (E), as a percentage of the whole area. AV, Avascular area; NV, Neovascularization area. Untreated OIR control (*n* = 6), PBS (*n* = 7), bevacizumab (*n* = 8), PEDF+bevacizumab (*n* = 4), PEDF (*n* = 6), anti‐rat VEGF (*n* = 6), PEDF+anti‐rat VEGF (*n* = 5). Mean and standard deviation are shown, *p < 0.05, **p < 0.001, ***p < 0.0001, *n* = number of eyes. Statistical significances against the OIR control are indicated in black, against the PBS control in gray.

Figure 4A displays a representative flatmount image of a P17 rat eye with OIR. An area with pronounced vessel obliteration and neovascularization is outlined in red. A magnified image of this area is provided in Figure 4B. The principle of the flatmount analysis is illustrated in Figure 4C: the whole area (white framed), avascular (red) and neovascularization (yellow) areas were manually selected on the flatmount images and used for the analysis. As depicted in Figure 4D, the neovascularization area of the groups treated with PEDF, PEDF + bevacizumab, PEDF + anti‐rat VEGF, bevacizumab, and anti‐rat VEGF was significantly reduced compared to the neovascularization area of the untreated OIR control (ANOVA, ***p < 0.0001, indicated in black) and PBS control (ANOVA, *p < 0.05, indicated in gray). The neovascularization area of the OIR and PBS controls showed no significant difference compared to each other (ANOVA, *p* > 0.05). There was also a statistically significant reduction of the avascular area, meaning a significant reduction of vessel obliteration for all treatment groups, compared to the untreated OIR (Figure 4E, ANOVA, **p < 0.001, *p < 0.05, indicated in black) and the PBS (ANOVA, *p < 0.05, indicated in gray) controls. There was no significant difference when the untreated OIR control was compared to the PBS control (*p* > 0.05**)**.

Representative in vivo (SLO/FA) images and the corresponding flatmount images of the same eyes for each group (untreated control, PBS control, PEDF protein, PEDF protein+bevacizumab, PEDF protein + anti‐rat VEGF, bevacizumab and anti‐rat VEGF) are shown in Figure S1. While the flatmounts allowed the imaging of the whole retina, the SLO/FA analysis allowed only a limited view of the central part of the retina. In our rat model, the neovascularized and AV were mostly located in the periphery of the retina. Note the reduction of the artery tortuosity in the eyes treated with PEDF protein alone or in combination with VEGF antagonists.

#### 3.3.2 Analysis of the vessel tortuosity on flatmount images

**FIGURE 5:**
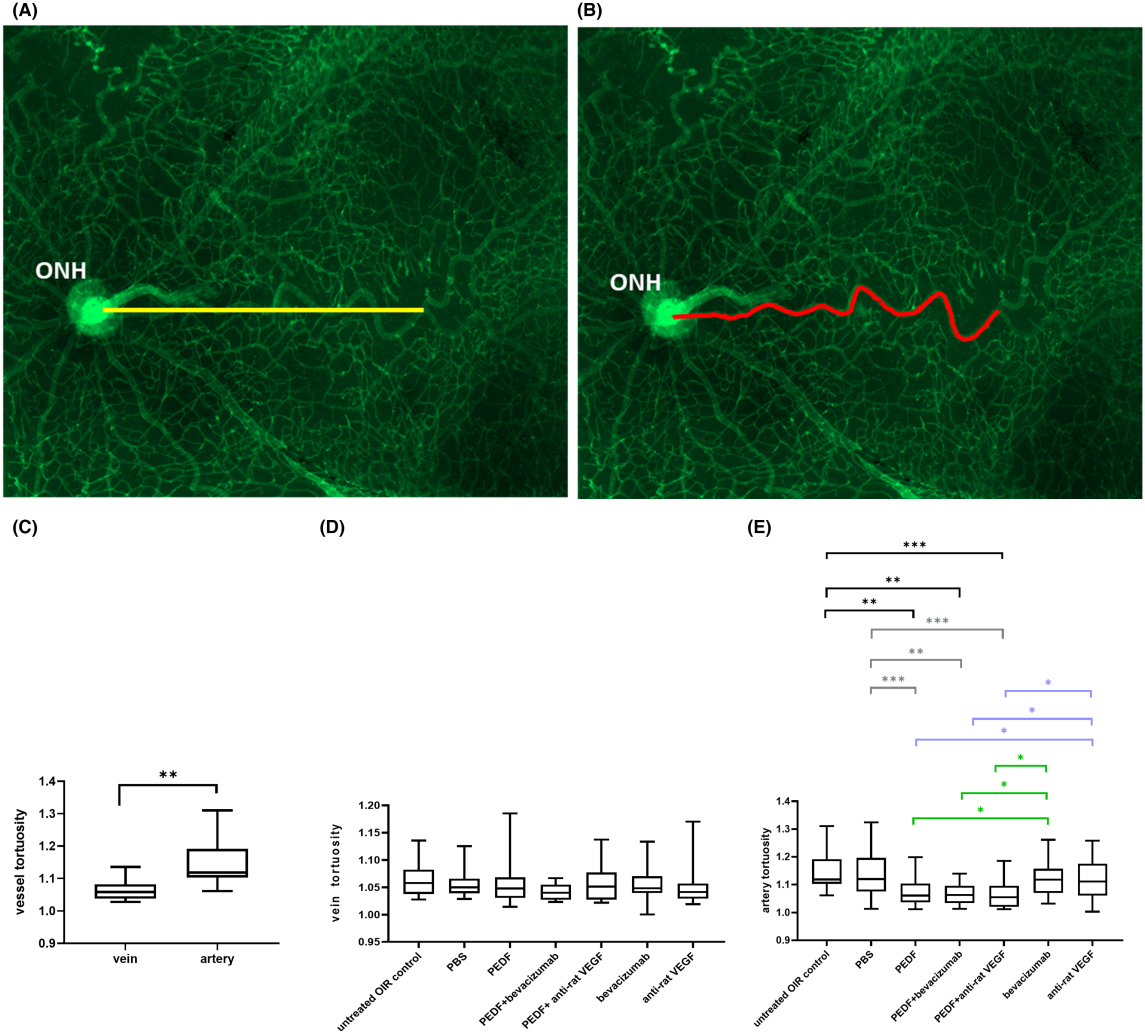
Vessel tortuosity analysis on flatmount images. (A) The yellow line represents the distance of a retinal vessel from the optic nerve head (ONH) to the point of its first bifurcation. (B) The red line indicates the length from the ONH to the first bifurcation point of the vessel analyzed in (A). The tortuosity of a vessel is defined as the ratio of the length (B) and distance (A) of the vessel. (C) Direct statistical comparison of the tortuosity of the veins and arteries in the untreated OIR control animal eyes (*n* = 6). (D) ANOVA analysis of the vein tortuosity and e artery tortuosity. Untreated OIR control (*n* = 6), PBS (*n* = 7), bevacizumab (*n* = 8), PEDF+bevacizumab (*n* = 4), PEDF (*n* = 6), anti‐rat VEGF (*n* = 6), PEDF+anti‐rat VEGF (*n* = 5). Mean and standard deviation are shown *p < 0.05, **p < 0.001, ***p < 0.0001, *n* = number of eyes. Statistically significant differences against the untreated OIR control are indicated in black, against the PBS control in gray, against bevacizumab in green and against anti‐rat VEGF in blue.

Figure 5A,B illustrates the principle of vessel tortuosity analysis on flatmount images. In Figure 5A, the yellow line denotes the distance of a retinal vessel from the ONH to its first bifurcation point. In Figure 5B, the red line represents the length from the ONH to the first bifurcation point of the vessel shown in (a). Vessel tortuosity is defined as the ratio of the length (b) to the distance (a) of the vessel. As depicted in Figure 5C, the overall artery tortuosity was significantly higher compared to the overall vein tortuosity in the untreated control group (Student's t‐test, *p* < 0.001). None of the treatment modalities affected vein tortuosity (Figure 5D, ANOVA, p > 0.05). Intriguingly, treatment with PEDF protein alone or in combination with VEGF antagonists significantly reduced artery tortuosity compared to untreated and PBS controls, as well as compared to groups treated only with VEGF antagonists (Figure 5E, ANOVA, ***p < 0.0001, **p < 0.001, *p < 0.05).

#### 3.3.3 Analysis of the vessel diameter on flatmounts and on OCT images

**FIGURE 6:**
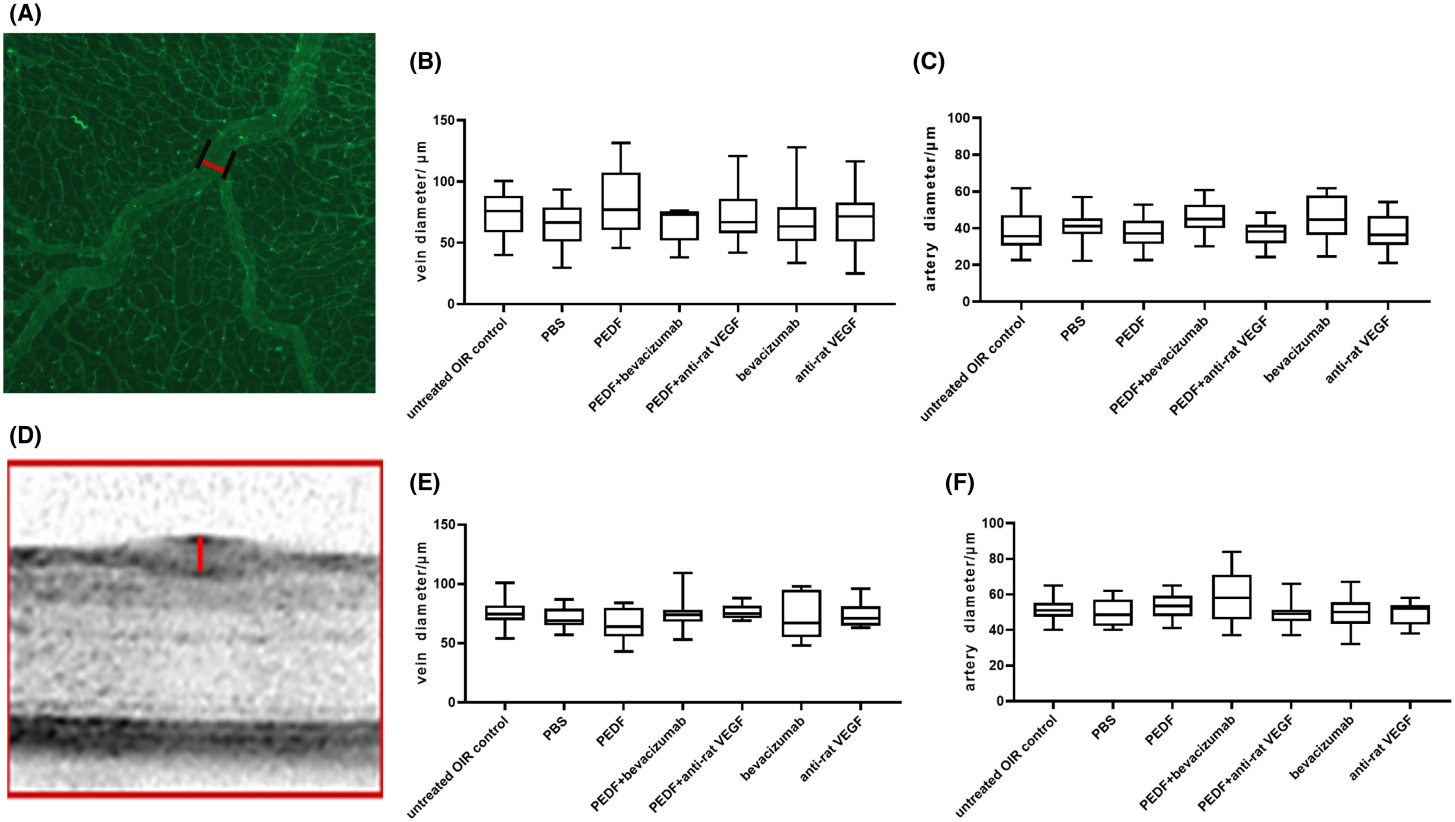
Vessel diameter analysis on flatmount and OCT images. (A) The diameters (red line) were measured at the first bifurcation point of the vessels perpendicular to the vessel walls (black lines) on flatmount images. (B) ANOVA analysis of the vein diameter and (C) artery diameter measurements. Untreated OIR control (*n* = 6), PBS (*n* = 7), bevacizumab (*n* = 8), PEDF + bevacizumab (*n* = 4), PEDF (*n* = 6), anti‐rat VEGF (*n* = 6), PEDF + anti‐rat VEGF (*n* = 5). (D) Principle of the vessel diameter measurement on OCT images (red line). (E) ANOVA analysis of the vein diameter and (F)artery diameter measurements. Untreated OIR control (*n* = 8), PBS (*n* = 7), bevacizumab (*n* = 8), PEDF + bevacizumab (*n* = 8), PEDF (*n* = 7), anti‐rat VEGF (*n* = 5), PEDF + anti‐rat VEGF (*n* = 5). Mean ± standard deviation are shown, *n* = number of eyes.

Flatmount (Figure 6A–C) and OCT images (Figure 6D–F) analysis revealed that none of the used treatments significantly affected the artery or vein diameters (ANOVA, *p* > 0.05).

## 4 DISCUSSION

Several common retinal diseases that cause blindness are characterized by pathological neovascularization accompanied by inflammation and neurodegeneration, including ROP, diabetic retinopathy, age‐related macular degeneration, and retinal vein occlusion. The current treatment strategies for these diseases have limited benefits. Thus, safer and more effective alternative approaches are required.^29^ Although several studies have shown that PEDF has multiple physiological effects, including anti‐angiogenic, anti‐inflammatory, antioxidant, and neuroprotective effects,^29^ PEDF has not been used in clinical practice due to its limited tissue penetrability and biological stability. However, the impact of proteins on the treatment of various conditions, including ocular diseases, has advanced significantly over the past few decades due to substantial breakthroughs in structural biochemistry, genetic engineering, formulation and delivery approaches^36^ making the use of PEDF protein‐based therapeutics interesting again.

In the present study, a PEDF protein safety study was performed in rat eyes by testing three doses (4, 20, and 100 μg/eye) at four different time points (days 1, 3, 7, and 14) after IVI. The eyes were investigated using histology and ERG for functional retinal testing. Cellular infiltration into the vitreous was observed predominantly 3 days after PEDF injection and may be caused by PEDF directly or by bacterial contamination, which cannot be completely excluded. Also, the fact that the protein is human may have stimulated the MC. Similar results were found after the IVI of Fc‐fragments into the vitreous of rats.^37^ Because cellular infiltrates disappeared nearly completely after seven and 14 days, these were not regarded as an adverse effect. The results of GFAP staining showed that the treatment with PEDF activated the Mueller glial cells/macrophages throughout the observation period. Because the Mueller cells did not change their shape and always extended longitudinally from the ILM to the OLM, this was considered mild activation. In a monkey study using bevacizumab at the same dose used in patients, the expression of GFAP was more prominent and even seen in astrocytes.^38^ Nevertheless, bevacizumab is used to treat numerous diseases in ophthalmology since many years. Therefore, and because astrocytes did not express GFAP in this study, the slightly enhanced expression of GFAP was also not considered an adverse effect. MC are resident macrophages and have several physiological functions, such as controlling neuronal cell production, neural migration, axonal growth, synaptogenesis, and angiogenesis.^39^ They also play an important role under pathological conditions by protecting the retina during infection, inflammation, trauma, ischemia, tumor, and neurodegeneration. MC have two modes of action: in the healthy retina, they are inactive (resting) and become active during an immune response. Resting and activated MC have distinct morphology and cell surface markers. Resting MC have a small soma and elongated ramified processes extending and retracting continuously. During the first activation step, the elongated ramified processes become more prominent in the cells. When MC recognize a pathogen or other inflammatory stimulus, they rapidly reach an active state, retract their processes, and become efficient mobile effector cells.^39^ The results of this study revealed that the MC were activated already 1–3 days after PEDF injection, clearly indicated by the enhanced number of cells with elongated ramified processes in the sections. However, it returned nearly to that of untreated retinas in both high dosage groups (20 and 100 μg PEDF) after 14 days. This indicates that the activation is restricted to a short period, even at a high PEDF dose, and is therefore also not regarded as an adverse effect, which was corroborated by the functional retinal testing using ERG (Figure 1).

The OIR model was first developed in the kitten by Ashton et al.,^40^ then extended to the rat^41^ and the mouse.^42^ Due to the similar features in the retina of OIR animals and in the ROP patients, the OIR model has been widely used in research involving ROP. The retinal angiogenesis in the OIR rat model is more similar to the pathology observed in ROP patients than in the mouse model.^34^, ^43^ Indeed, the avascular area (meaning vaso‐obliteration) is more peripheral in the rat OIR model and in human ROP, while it occurs primarily in the central retina in the murine OIR model. In the initial publication, by Penn et al.,^41^ the authors used albino rats and compared the occurrence of pre‐retinal neovascularization in animals exposed to cyclic high oxygen periods followed by room air periods versus a constant high oxygen period followed by room air. The animals were treated immediately after birth, and they found that the alternating hyperoxia‐hypoxia cycles better simulated the ROP features. Later, the alternating oxygen cyclic protocol was revised. The new protocol reached a higher incidence and severity of neovascularization.^44^ Although our group^45^ and others have reported that the lack of melanin causes impaired visual acuity, a lower number of ipsilaterally projecting retinal ganglion cells, defects in the optokinetic nystagmus, and a lower functional response,^46^ the vast majority of eye researchers still use the albino rat strain. In this study, we improved our previously established rat OIR model^28^ by using pigmented rats. The animals were exposed to 75% oxygen from P7 to P12, then moved to room air (similar to the conditions applied to premature babies), followed immediately by IVI of the different therapeutics. The read‐out was performed at P17. Our OIR model showed typical ROP characteristics such as vessel tortuosity, peripheral vessel obliteration, and neovascularization (Figure 4). This model was used in the present study to evaluate the effects of PEDF protein alone or combined with anti‐VEGF agents compared to anti‐VEGFs alone on pathological neovascularization and non‐neovascular remodeling characterized by tortuosity and dilation of the retinal vessels. Indeed, abnormalities of the retinal vessels are a clinically important finding of ischemic retinopathy in addition to pathological angiogenesis. Bevacizumab, an anti‐human VEGF drug, was used as a positive control for countering neovascularization as it is widely used in retinal vessel diseases.^16^, ^47^, ^48^ In addition, an anti‐rat VEGF drug was also used as a species‐specific positive control. A human PEDF protein was used in this study, the amino acid homology between human PEDF and rat PEDF is about 83%.^49^ A dose‐ranging study of human PEDF protein (24.5, 12.25, and 6.125 μg/eye) was conducted in the OIR rat model to find the dose with the highest efficacy. The dose of 6.125 μg PEDF protein/eye was shown to significantly reduce pathological neovascularization and vessel obliteration. Moreover, the hyaloid vessels and the overall retinal vessel density and tortuosity were also reduced (Figure 3A,B) and showed the closest resemblance to that in animals growing under normoxic conditions as described by Vahatupa et al.^34^ Thus, the dose of 6.125 μg PEDF protein/eye showed the most beneficial effects on the eyes after OIR and was used in the present study.

In our OIR rat model, the neovascularized and AV were mostly located in the periphery of the retina, as occurs in ROP patients. The flatmounts allowed the imaging of the entire retina, while the SLO/FA images only allowed a limited view and imaging of the central part of the retina (Figure 1). Therefore, accurate quantification of pathological retinal neovascularization and vessel obliteration could only be performed in flatmounts (Figure 4). However, SLO/FA can generally be used for a non‐invasive screening of therapeutic efficacy. We showed that treatment with PEDF protein alone or in combination with VEGF antagonists significantly suppressed pathological neovascularization and reduced vessel obliteration, as observed when using anti‐VEGF drugs alone compared to untreated and PBS controls (Figure 4).

The decrease in the avascular area, in addition to the inhibition of the formation of neovascular tufts in the retina of the OIR model, suggests that the use of PEDF protein could lead to a recovery from ischemia. Therefore, PEDF protein showed similar effects to the established anti‐VEGF agents, having the advantages of being a natural VEGF antagonist and potentially lacking the side effects of anti‐VEGF injections such as unfavorable neurodevelopment in the long term.^50^

Our results corroborate some effects of PEDF‐derived‐peptides investigated by Sheibani et al.,^23^ in a murine OIR model. They found that PEDF 336, a nonapeptide, can reduce the avascular area and pathological neovascularization in the OIR mouse retina. However, in contrast to our study performed in rats, Sheibani et al.^23^ found that bevacizumab did not reduce the avascular area nor neovascularization in their murine OIR model. From the aforementioned comparison, although mice and rats both belong to rodents, murine and rat VEGF depict different responses to bevacizumab, challenging the findings from Yu et al.,^51^ which emphasized that bevacizumab has poor affinity to murine VEGF.

In addition to the avascular and NV, artery, and vein tortuosities are also important parameters that illustrate retinal vasculature in the OIR rat model.^52^ The tortuosity has also been proved to be the early readout for neovascularization in OIR mouse models,^53^ which also simulate the characteristics of the intraretinal microvascular abnormality^54^ in diabetic retinopathy. For the quantification of the vessel tortuosity, some researchers did not differentiate veins from arteries,^55^ whereas others quantified artery and vein tortuosities separately.^56^ Hartnett et al.^56^ attested that only artery tortuosity increased in the OIR model while vein tortuosity remained statistically unchanged. In the present study, to find the appropriate method, vein, and artery tortuosities were quantified separately in the untreated group (Figure 5C). We found that artery tortuosity was significantly higher than vein tortuosity in the untreated OIR group, so vein and artery tortuosities were compared independently in the different treated groups. Interestingly, only the treatments with PEDF protein alone or in combination with an anti‐VEGF drug were able to significantly reduce artery tortuosity in our OIR rat retina compared to the untreated and PBS controls, as well as to the treatment with the anti‐VEGF agents (Figure 5D,E). Ergo, only the PEDF protein‐based therapy (i.e. PEDF protein alone or in combination with an anti‐VEGF drug) improved the quality of the retinal vasculature in OIR rats. The results of our tortuosity analysis differ from the study results of Hartnett et al.^56^ in which artery tortuosity was reduced after treatment with an anti‐rat VEGF. As we measured artery tortuosity 3 days later than Hartnett et al., one possible explanation for this discrepancy could be that the anti‐rat VEGF may reduce artery tortuosity in the early stage in the OIR rat model, but this reduction does not last. Additionally, the authors used another experimental protocol and a rat neutralizing antibody against VEGF^164^ at a different concentration (50 ng/μL).

Another clinical hallmark of acute ROP is the dilation of the retinal vasculature.^57^ Therefore, the effect of the different treatments on vessel diameter was also investigated. Considering that perfusion alteration may influence vessel diameter, this was measured in vivo using SLO/OCT and ex vivo on flatmounts. In addition, as the thicknesses of the veins and arteries are statistically different in rats,^35^ their diameters were measured independently both in vivo and ex vivo. Neither PEDF protein alone nor anti‐VEGF agents nor their combination affected the vessel diameters with (in vivo) or without (ex vivo) perfusion in our study (Figure 6). This finding contrasts with the result from Hartnett et al. suggesting that treatment with antibodies against VEGF reduced venous dilation.^56^ However, as mentioned earlier, the discrepancy can be explained due to differences in the protocols and analysis time points.

Although PEDF has been found to be the most potent natural, endogenous inhibitor of neovascularization, its application is still restricted because of its instability and short half‐life.^58^ It is known that the major challenge of using proteins or peptides is their poor in vivo stability, retention, and inactivation by the immune system or by the action of proteolytic enzymes.^59^ Rapid elimination leads to frequent and excessive administration, which is impractical and causes non‐specific toxicity. Thus, controlled release, which will make it accumulate to effective levels and be metabolized with minimum toxicity and without intolerable adverse effects, becomes particularly important. A crucial strategy for controlled release is the use of polymers.^60^ Conjugation of proteins with polymers reduces recognition by the immune system and decreases the clearance rate.^59^ Currently, polyethylene glycol (PEG) is one of the most widely used polymers for the modification of protein therapeutics, with many applications from industrial manufacturing to medicine.^61^ Because it is inert, inexpensive, and has low toxicity and increased solubility, PEG has been approved by the FDA for drug modification for several years.^62^ It has been shown that the half‐life of recombinant PEDF is 4.5 h, whereas the half‐life of modified PEGylated‐PEDF is 78 h.^58^ However, it has also been demonstrated that PEG can induce CNV,^63^ which is obviously not desirable for the treatment of ROP. Therefore, we propose delivering PEDF protein by using an implant similar to the FDA‐approved port delivery system for ranibizumab (Susvimo, Roche, Genentech), which allows as few as two treatments per year instead of a monthly injection.

To conclude, a single IVI of PEDF protein is safe and does not induce adverse effects in rat eyes even at a high dose (100 μg). PEDF protein therapy alone or in combination with VEGF antagonists significantly reduced pathological neovascularization and vessel obliteration in the rat OIR model, as did the anti‐VEGF agents alone. Additionally, we showed that treatments with PEDF protein alone or in combination with VEGF antagonists significantly reduced artery tortuosity, indicating an improvement in the retinal vasculature's quality. The use of VEGF antagonists alone did not affect vessel tortuosity. Therefore, PEDF protein alone or in combination with a VEGF antagonist is an alternative to anti‐VEGF agents for ROP. According to the results of this study and the latest international classification of ROP in 2021,^64^ PEDF protein therapy alone or in combination with anti‐VEGF could keep ROP below stage 3 by suppressing pathological neovascularization and reducing the avascular area as well as artery tortuosity.

## AUTHOR CONTRIBUTIONS

SZ, AVT, SK, SSK and SJS wrote the manuscript, SZ, AVT, KW, US, SSK and SJS conducted the experiments. SZ and AVT acquired and analyzed the data. US, SK and SJS designed the study. All authors were involved in drafting and revising the manuscript.

## CONFLICT OF INTEREST STATEMENT

SZ, AT, SSK, KW, SK and SJS have no competing interests to declare that are relevant to the content of this article. US is holder of a patent.

## Supporting information


Figure S1.

## Data Availability

The data that support the findings of this study are available in the methods of this article.

## References

[1] Khadawardi E , Al Hazzani F . Oxygen saturation and outcomes in preterm infants the BOOST II United Kingdom, Australia, and New Zealand collaborative groups. J Clin Neonatol. 2013;2:73‐75. 24049747 10.4103/2249-4847.116404 PMC3775139

[2] Fleck BW , Williams C , Juszczak E , et al. An international comparison of retinopathy of prematurity grading performance within the benefits of oxygen saturation targeting II trials. Eye (Lond). 2018;32:74‐80. 28752837 10.1038/eye.2017.150 PMC5669461

[3] Ashton N . Oxygen and the retinal blood vessels. Trans Ophthalmol Soc U K (1962). 1980;100:359‐362. 6171068

[4] Zhang RH , Liu YM , Dong L , et al. Prevalence, years lived with disability, and time trends for 16 causes of blindness and vision impairment: findings highlight retinopathy of prematurity. Front Pediatr. 2022;10:735335. 35359888 10.3389/fped.2022.735335 PMC8962664

[5] Campbell K . Intensive oxygen therapy as a possible cause of retrolental fibroplasia; a clinical approach. Med J Aust. 1951;2:48‐50. 14874698

[6] Bashinsky AL . Retinopathy of prematurity. N C Med J. 2017;78:124‐128. 28420777 10.18043/ncm.78.2.124

[7] Hartnett ME , Penn JS . Mechanisms and management of retinopathy of prematurity. N Engl J Med. 2012;367:2515‐2526. 23268666 10.1056/NEJMra1208129 PMC3695731

[8] Dai C , Webster KA , Bhatt A , Tian H , Su G , Li W . Concurrent physiological and pathological angiogenesis in retinopathy of prematurity and emerging therapies. Int J Mol Sci. 2021;22:4809. 34062733 10.3390/ijms22094809 PMC8124946

[9] Multicenter trial of cryotherapy for retinopathy of prematurity: preliminary results. Cryotherapy for retinopathy of prematurity cooperative group. Pediatrics. 1988;81:697‐706. 2895910

[10] Good WV , Early Treatment for Retinopathy of Prematurity Cooperative, G . Final results of the early treatment for retinopathy of prematurity (ETROP) randomized trial. Trans Am Ophthalmol Soc. 2004;102:233‐248. discussion 248‐250. 15747762 PMC1280104

[11] International Committee for the Classification of Retinopathy of, P . The international classification of retinopathy of prematurity revisited. Arch Ophthalmol. 2005;123:991‐999. 16009843 10.1001/archopht.123.7.991

[12] Ng EY , Connolly BP , McNamara JA , Regillo CD , Vander JF , Tasman W . A comparison of laser photocoagulation with cryotherapy for threshold retinopathy of prematurity at 10 years: part 1. Visual function and structural outcome. Ophthalmology. 2002;109:928‐934. discussion 935. 11986099 10.1016/s0161-6420(01)01017-x

[13] Andersen CC , Phelps DL . Peripheral retinal ablation for threshold retinopathy of prematurity in preterm infants. Cochrane Database Syst Rev. 2000;1999:CD001693. 10796444 10.1002/14651858.CD001693 PMC8406950

[14] Casteels I , Cassiman C , Van Calster J , Allegaert K . Educational paper: retinopathy of prematurity. Eur J Pediatr. 2012;171:887‐893. 22052209 10.1007/s00431-011-1610-7

[15] Sharma A , Shetty A , Reddy Y . Bevacizumab in retinopathy of prematurity: concerns and adverse effects. Nepal J Ophthalmol. 2020;12:298‐307. 33978625 10.3126/nepjoph.v12i2.28993

[16] Stahl A , Lepore D , Fielder A , et al. Ranibizumab versus laser therapy for the treatment of very low birthweight infants with retinopathy of prematurity (RAINBOW): an open‐label randomised controlled trial. Lancet. 2019;394:1551‐1559. 31522845 10.1016/S0140-6736(19)31344-3 PMC12316478

[17] Dai C , Xiao J , Wang C , Li W , Su G . Neurovascular abnormalities in retinopathy of prematurity and emerging therapies. J Mol Med (Berl). 2022;100:817‐828. 35394143 10.1007/s00109-022-02195-2 PMC9205172

[18] Tan JJ , Cai CL , Shrier EM , et al. Ocular adverse effects of intravitreal bevacizumab are potentiated by intermittent hypoxia in a rat model of oxygen‐induced retinopathy. J Ophthalmol. 2017;2017:4353129. 28770109 10.1155/2017/4353129 PMC5523466

[19] Morin J , Luu TM , Superstein R , et al. Neurodevelopmental outcomes following bevacizumab injections for retinopathy of prematurity. Pediatrics. 2016;137:e20153218. 27244705 10.1542/peds.2015-3218

[20] Ortiz‐Seller A , Martorell P , Barranco H , Pascual‐Camps I , Morcillo E , Ortiz JL . Comparison of different agents and doses of anti‐vascular endothelial growth factors (aflibercept, bevacizumab, conbercept, ranibizumab) versus laser for retinopathy of prematurity: a network meta‐analysis. Surv Ophthalmol. 2024;69:585‐605. 38432359 10.1016/j.survophthal.2024.02.005

[21] Barnstable CJ , Tombran‐Tink J . Neuroprotective and antiangiogenic actions of PEDF in the eye: molecular targets and therapeutic potential. Prog Retin Eye Res. 2004;23:561‐577. 15302351 10.1016/j.preteyeres.2004.05.002

[22] He X , Cheng R , Benyajati S , Ma JX . PEDF and its roles in physiological and pathological conditions: implication in diabetic and hypoxia‐induced angiogenic diseases. Clin Sci (Lond). 2015;128:805‐823. 25881671 10.1042/CS20130463 PMC4557399

[23] Sheibani N , Zaitoun IS , Wang S , et al. Inhibition of retinal neovascularization by a PEDF‐derived nonapeptide in newborn mice subjected to oxygen‐induced ischemic retinopathy. Exp Eye Res. 2020;195:108030. 32272114 10.1016/j.exer.2020.108030 PMC7282953

[24] Zhang SX , Wang JJ , Gao G , Parke K , Ma JX . Pigment epithelium‐derived factor downregulates vascular endothelial growth factor (VEGF) expression and inhibits VEGF‐VEGF receptor 2 binding in diabetic retinopathy. J Mol Endocrinol. 2006;37:1‐12. 16901919 10.1677/jme.1.02008

[25] Duh EJ , Yang HS , Suzuma I , et al. Pigment epithelium‐derived factor suppresses ischemia‐induced retinal neovascularization and VEGF‐induced migration and growth. Invest Ophthalmol Vis Sci. 2002;43:821‐829. 11867604

[26] Amaral J , Becerra SP . Effects of human recombinant PEDF protein and PEDF‐derived peptide 34‐mer on choroidal neovascularization. Invest Ophthalmol Vis Sci. 2010;51:1318‐1326. 19850839 10.1167/iovs.09-4455 PMC2836227

[27] Sheibani N , Wang S , Darjatmoko SR , et al. Novel anti‐angiogenic PEDF‐derived small peptides mitigate choroidal neovascularization. Exp Eye Res. 2019;188:107798. 31520600 10.1016/j.exer.2019.107798 PMC7032632

[28] Semkova I , Kreppel F , Welsandt G , et al. Autologous transplantation of genetically modified iris pigment epithelial cells: a promising concept for the treatment of age‐related macular degeneration and other disorders of the eye. Proc Natl Acad Sci USA. 2002;99:13090‐13095. 12239351 10.1073/pnas.202486199 PMC130591

[29] Fan R , Su L , Zhang H , et al. Enhanced therapeutic effect of PEDF‐loaded mesenchymal stem cell‐derived small extracellular vesicles against oxygen‐induced retinopathy through increased stability and penetrability of PEDF. J Nanobiotechnology. 2023;21:327. 37684667 10.1186/s12951-023-02066-z PMC10492320

[30] Xi L , Tikhonovich M , Biesemeier A , Julien‐Schraermeyer S , Schraermeyer U , Tschulakow AV . Pigment epithelium‐derived factor protects retinal neural cells and prevents pathological angiogenesis in an ex vivo ischemia model. Oxidative Med Cell Longev. 2022;2022:4199394. 10.1155/2022/4199394 PMC9410835 36035211

[31] Tschulakow AV , Xi L , Schraermeyer U , Julien‐Schraermeyer S . Pigment epithelium‐derived factor (PEDF) based therapy induced photoreceptor survival by stabilising the neovessels in a VEGF overexpression CNV rat model. Invest Ophthalmol Vis Sci. 2023;64:1133. 10.1096/fj.202501704RR PMC12510281 41065753

[32] Liu S , Biesemeier AK , Tschulakow AV , Thakkar HV , Julien‐Schraermeyer S , Schraermeyer U . A new rat model of treatment‐naive quiescent choroidal neovascularization induced by human VEGF165 overexpression. Biol Open. 2020;9:bio048736. 32086250 10.1242/bio.048736 PMC7295592

[33] Tual‐Chalot S , Allinson KR , Fruttiger M , Arthur HM . Whole mount immunofluorescent staining of the neonatal mouse retina to investigate angiogenesis in vivo. J Vis Exp. 2013;9:e50546. 10.3791/50546 PMC3732076 23892721

[34] Vahatupa M , Jaaskelainen N , Cerrada‐Gimenez M , et al. Oxygen‐induced retinopathy model for ischemic retinal diseases in rodents. J Vis Exp. 2020;16:163. 10.3791/61482 33016936

[35] Pannarale L , Onori P , Ripani M , Gaudio E . Precapillary patterns and perivascular cells in the retinal microvasculature. A scanning electron microscope study. J Anat. 1996;188(Pt 3):693‐703. 8763486 PMC1167497

[36] Mandal A , Pal D , Agrahari V , Trinh HM , Joseph M , Mitra AK . Ocular delivery of proteins and peptides: challenges and novel formulation approaches. Adv Drug Deliv Rev. 2018;126:67‐95. 29339145 10.1016/j.addr.2018.01.008 PMC5995646

[37] Taubitz T , Steinbrenner LP , Tschulakow AV , Biesemeier A , Julien‐Schraermeyer S , Schraermeyer U . Effects of intravitreally injected fc fragment on rat eyes. Graefes Arch Clin Exp Ophthalmol. 2016;254:2401‐2409. 27752777 10.1007/s00417-016-3511-y

[38] Heiduschka P , Fietz H , Hofmeister S , et al. Penetration of bevacizumab through the retina after intravitreal injection in the monkey. Invest Ophthalmol Vis Sci. 2007;48:2814‐2823. 17525217 10.1167/iovs.06-1171

[39] Endo Y , Asanuma D , Namiki S , et al. Quantitative modeling of regular retinal microglia distribution. Sci Rep. 2021;11:22671. 34811401 10.1038/s41598-021-01820-3 PMC8608893

[40] Ashton N , Ward B , Serpell G . Effect of oxygen on developing retinal vessels with particular reference to the problem of retrolental fibroplasia. Br J Ophthalmol. 1954;38:397‐432. 13172417 10.1136/bjo.38.7.397 PMC1324374

[41] Penn JS , Tolman BL , Lowery LA . Variable oxygen exposure causes preretinal neovascularization in the newborn rat. Invest Ophthalmol Vis Sci. 1993;34:576‐585. 8449677

[42] Smith LE , Wesolowski E , McLellan A , et al. Oxygen‐induced retinopathy in the mouse. Invest Ophthalmol Vis Sci. 1994;35:101‐111. 7507904

[43] Kim CB , D'Amore PA , Connor KM . Revisiting the mouse model of oxygen‐induced retinopathy. Eye Brain. 2016;8:67‐79. 27499653 10.2147/EB.S94447 PMC4975545

[44] Penn JS , Rajaratnam VS . Inhibition of retinal neovascularization by intravitreal injection of human rPAI‐1 in a rat model of retinopathy of prematurity. Invest Ophthalmol Vis Sci. 2003;44:5423‐5429. 14638747 10.1167/iovs.02-0804

[45] Heiduschka P , Schraermeyer U . Comparison of visual function in pigmented and albino rats by electroretinography and visual evoked potentials. Graefes Arch Clin Exp Ophthalmol. 2008;246:1559‐1573. 18654793 10.1007/s00417-008-0895-3

[46] Galindo‐Romero C , Norte‐Munoz M , Gallego‐Ortega A , et al. The retina of the lab rat: focus on retinal ganglion cells and photoreceptors. Front Neuroanat. 2022;16:994890. 36213609 10.3389/fnana.2022.994890 PMC9538360

[47] Kim EJ , Lin WV , Rodriguez SM , Chen A , Loya A , Weng CY . Treatment of diabetic macular edema. Curr Diab Rep. 2019;19:68. 31359157 10.1007/s11892-019-1188-4

[48] Kailar RS , Kuo BL , Perkins SW , Singh RP . Long‐term outcomes in early versus limited response to anti‐VEGF treatment for retinal vein occlusion. Ophthalmol Retina. 2024;8:55‐61. 37595685 10.1016/j.oret.2023.08.005

[49] Tombran‐Tink J , Aparicio S , Xu X , et al. PEDF and the serpins: phylogeny, sequence conservation, and functional domains. J Struct Biol. 2005;151:130‐150. 16040252 10.1016/j.jsb.2005.05.005

[50] Tsai AS , Chou HD , Ling XC , et al. Assessment and management of retinopathy of prematurity in the era of anti‐vascular endothelial growth factor (VEGF). Prog Retin Eye Res. 2022;88:101018. 34763060 10.1016/j.preteyeres.2021.101018

[51] Yu L , Wu X , Cheng Z , et al. Interaction between bevacizumab and murine VEGF‐A: a reassessment. Invest Ophthalmol Vis Sci. 2008;49:522‐527. 18234994 10.1167/iovs.07-1175

[52] Kim Y , Hong HK , Park JR , et al. Oxygen‐induced retinopathy and choroidopathy: in vivo longitudinal observation of vascular changes using OCTA. Invest Ophthalmol Vis Sci. 2018;59:3932‐3942. 30073364 10.1167/iovs.18-24320

[53] Scott A , Powner MB , Fruttiger M . Quantification of vascular tortuosity as an early outcome measure in oxygen induced retinopathy (OIR). Exp Eye Res. 2014;120:55‐60. 24418725 10.1016/j.exer.2013.12.020

[54] Sayin N , Kara N , Pekel G . Ocular complications of diabetes mellitus. World J Diabetes. 2015;6:92‐108. 25685281 10.4239/wjd.v6.i1.92 PMC4317321

[55] Guaiquil VH , Hewing NJ , Chiang MF , Rosenblatt MI , Chan RV , Blobel CP . A murine model for retinopathy of prematurity identifies endothelial cell proliferation as a potential mechanism for plus disease. Invest Ophthalmol Vis Sci. 2013;54:5294‐5302. 23833070 10.1167/iovs.12-11492 PMC3738219

[56] Hartnett ME , Martiniuk D , Byfield G , Geisen P , Zeng G , Bautch VL . Neutralizing VEGF decreases tortuosity and alters endothelial cell division orientation in arterioles and veins in a rat model of ROP: relevance to plus disease. Invest Ophthalmol Vis Sci. 2008;49:3107‐3114. 18378573 10.1167/iovs.08-1780 PMC2459334

[57] Fulton AB , Akula JD , Mocko JA , et al. Retinal degenerative and hypoxic ischemic disease. Doc Ophthalmol. 2009;118:55‐61. 18483822 10.1007/s10633-008-9127-8 PMC2629502

[58] Bai YJ , Huang LZ , Xu XL , et al. Polyethylene glycol‐modified pigment epithelial‐derived factor: new prospects for treatment of retinal neovascularization. J Pharmacol Exp Ther. 2012;342:131‐139. 22495066 10.1124/jpet.112.192575

[59] Abuchowski A , McCoy JR , Palczuk NC , van Es T , Davis FF . Effect of covalent attachment of polyethylene glycol on immunogenicity and circulating life of bovine liver catalase. J Biol Chem. 1977;252:3582‐3586. 16907

[60] Qiu LY , Bae YH . Polymer architecture and drug delivery. Pharm Res. 2006;23:1‐30. 16392022 10.1007/s11095-005-9046-2

[61] Pai SS , Tilton RD , Przybycien TM . Poly(ethylene glycol)‐modified proteins: implications for poly(lactide‐co‐glycolide)‐based microsphere delivery. AAPS J. 2009;11:88‐98. 19199044 10.1208/s12248-009-9081-8 PMC2664882

[62] Harris JM , Chess RB . Effect of pegylation on pharmaceuticals. Nat Rev Drug Discov. 2003;2:214‐221. 12612647 10.1038/nrd1033

[63] Lyzogubov VV , Tytarenko RG , Liu J , Bora NS , Bora PS . Polyethylene glycol (PEG)‐induced mouse model of choroidal neovascularization. J Biol Chem. 2011;286:16229‐16237. 21454496 10.1074/jbc.M110.204701 PMC3091230

[64] Chiang MF , Quinn GE , Fielder AR , et al. International classification of retinopathy of prematurity, third edition. Ophthalmology. 2021;128:e51‐e68. 34247850 10.1016/j.ophtha.2021.05.031 PMC10979521

