# Supplementary figures and images for: Reduction of pathological retinal neovascularization, vessel obliteration, and artery tortuosity by PEDF protein in an oxygen‐induced ischemic retinopathy rat model
Source: FASEB Bioadv. 2024 Jul 19;6(9):311–26. doi: 10.1096/fba.2024-00059 (PMC11467744; doi:10.1096/fba.2024-00059)

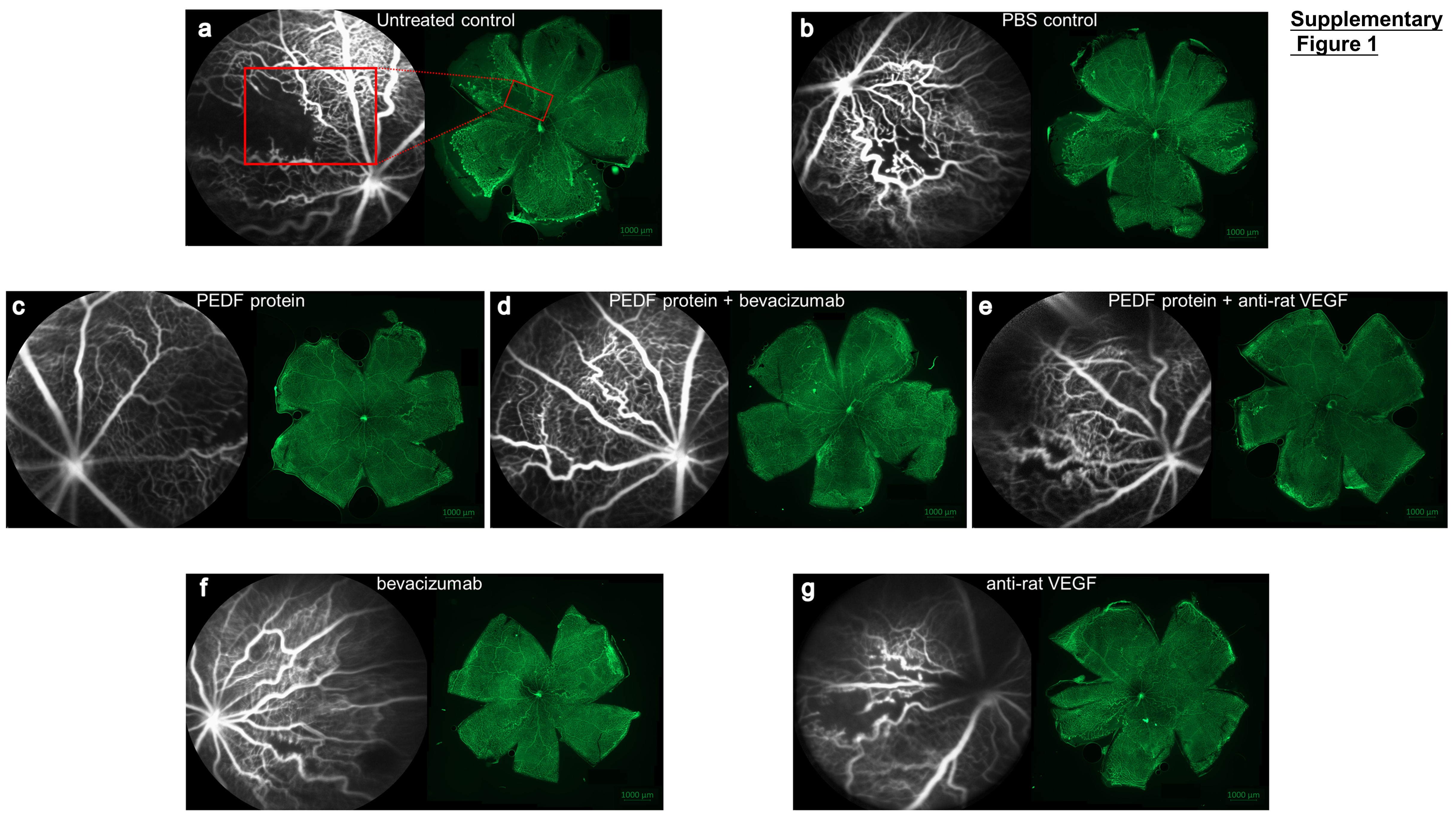

Supplement: Supplementary file 1 — Figure S1. [file FBA2-6-311-s001.tif]
